# A novel integrated biomarker index for the assessment of hematological responses in MPNs during treatment with hydroxyurea and interferon‐alpha2

**DOI:** 10.1002/cam4.5285

**Published:** 2022-10-17

**Authors:** Marc J. B. Dam, Rasmus K. Pedersen, Trine A. Knudsen, Morten Andersen, Christina Ellervik, Morten Kranker Larsen, Lasse Kjær, Vibe Skov, Hans C. Hasselbalch, Johnny T. Ottesen

**Affiliations:** ^1^ Centre for Mathematical Modeling ‐ Human Health and Disease (COMMAND), IMFUFA, Department of Science and Environment Roskilde University Roskilde Denmark; ^2^ Department of Haematology Zealand University Hospital Roskilde Denmark; ^3^ Department of Research, Production, Innovation, Region Zealand Sorø Denmark; ^4^ Department of Pathology Harvard Medical School Boston Massachusetts USA

**Keywords:** combination therapy, essential thrombocythemia, hydroxyurea, interferon‐alpha2, MPN, myelofibrosis, novel integrated biomarker index, polycythemia vera, treatment responses

## Abstract

**Background:**

Conventional cytoreductive therapy for patients with chronic Philadelphia‐negative myeloproliferative neoplasms (MPNs) includes hydroxyurea (HU), interferon‐alpha2 (IFN), and anagrelide. HU is worldwide the most used cytoreductive agent, which lowers elevated blood cell counts within days in the large majority of patients. However, some patients may experience rebound cytosis when HU is reduced due to cytopenia, thereby potentially giving rise to fluctuating cell counts during therapy. Such rapid oscillations may be harmful and potentially elicit thrombosis. Treatment with IFN gradually lowers elevated cell counts within weeks and when the dosage is reduced, the cell counts do not rapidly increase but are sustained within the normal range in the large majority of patients. Conventional hematological response criteria are among others based upon single absolute cell count values and do not take into account the relative decreases toward normal for each cell count.

**Materials, Methods & Results:**

Using serial data from the Danish DALIAH trial, we herein describe a novel integrated biomarker index for the assessment of hematological and molecular (*JAK2V617F*) responses in patients with MPNs during treatment with IFN or HU.

**Discussion:**

This novel tool convincingly displays the superiority of IFN versus HU in normalizing elevated cell counts. Our results need to be validated in larger studies but already now call for studies of the safety and efficacy of combination therapy during the initial treatment of patients with MPNs.

## Graphical abstract

A Novel Integrated Biomarker Index for the Assessment of Hematological Responses during Treatment with Hydroxyurea and Interferon‐alpha2. This novel tool convincingly displays the superiority of interferon‐alpha2 versus hydroxyurea in normalizing elevated cell counts.

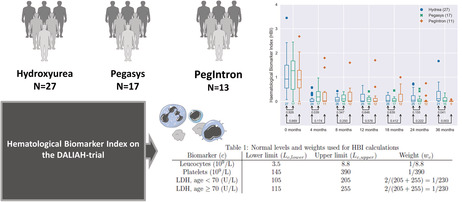

## 1 INTRODUCTION

The classic Philadelphia‐negative chronic myeloproliferative neoplasms (MPNs) encompass essential thrombocythemia (ET), polycythemia vera (PV), and primary myelofibrosis (PMF).^1^ Prior to the MPN diagnosis, many patients may experience thromboembolic events with concurrent elevated cell counts for several years due to undiagnosed MPNs.^2^, ^3^, ^4^, ^5^, ^6^ The MPNs are characterized by so‐called driver mutations in the following genes: *JAK2*, *CALR*, and *MPL. JAK2V617F* is the most frequent mutation being present in virtually all patients with PV and half of those with ET and PMF. Mutated *CALR* is found in approximately 50% of ET and PMF patients negative for the *JAK2V617F*‐mutation, respectively.^7^, ^8^, ^9^, ^10^, ^11^, ^12^, ^13^, ^14^ Additional mutations (e.g., *DNMT3A*, *ASXL1*, and *TET2*) are frequently recorded in the more advanced disease stages with severe myelofibrosis.^15^

Worldwide the most used cytoreductive agent in MPNs is hydroxyurea (HU)—a DNA‐synthesis inhibitor^16^, ^17^—that has raised concern in regard to its leukemogenic potential.^17^ Thus, some studies have shown that long‐term exposure to HU (> 10 years) may be associated with an increased risk of developing acute myelogenous leukemia (AML) or myelodysplastic syndrome (MDS), the latter with an inherently high risk of leukemic transformation.^18^, ^19^ HU does not target the malignant clone and accordingly does not correct the aberrant cellular machinery in MPNs. The cell counts rise to pretreatment levels within days following discontinuation of HU, highlighting that this agent does not influence the basic molecular aberrations that give rise to clonal expansion.

For about three decades, interferon‐alpha2 (IFN) has been used in the treatment of MPNs. Several studies have shown that IFN is safe and normalizes elevated cell counts efficiently.^20^, ^21^, ^22^ Prolonged treatment (about 5 years) with IFN may lead to polyclonal hematopoiesis, bone marrow normalization, and low‐burden *JAK2V617F* in a subset of patients, with continued effect even after 2–3 years following discontinuation of IFN.^23^, ^24^, ^25^, ^26^ These highly encouraging results have powered novel treatment goals of achieving “Minimal Residual Disease” (MRD).^27^

A major clinical challenge in MPN‐treatment is among others that the thrombosis risk is still substantial despite treatment with aspirin and in high‐risk patients cytoreductive therapy as well.^28^, ^29^, ^30^, ^31^, ^32^ Of note, the thrombosis risk for both venous and arterial thrombosis is most pronounced within the first 3 months after the MPN diagnosis.^30^ Most recently, we have shown that HU does not induce a sustained normalization of elevated cell counts or decrease in the *JAK2V617F* allele burden,^33^ which may contribute to the increased risk of thrombosis despite cytoreductive treatment.^28^, ^29^, ^30^, ^31^, ^32^ Most recent studies have shown that IFN is not superior to HU in terms of normalizing elevated cell counts after 12 and 24 months, however, IFN convincingly displays superiority after 36 months in regard to the potential of inducing major molecular remissions as assessed by a decline in the *JAK2V617F* allele burden.^22^, ^34^, ^35^ In these and previous studies, assessment of clinicohematological responses has been performed according to conventional international response criteria.^36^, ^37^ Accordingly, the biochemical responses for the leukocyte and platelet counts are based upon absolute values within the normal range. During treatment with, for example, HU and IFN, some patients achieve a normal leukocyte count but still have an elevated platelet count and others achieve a normal platelet count but still have an elevated leukocyte count. The ranges above normal may vary considerably. Thus, it might be anticipated that a patient with a normal leukocyte count (e.g., in the upper normal range) but still an elevated platelet count of e.g., 800 × 10^9^/L might have a response profile, which is inferior to the patient with a leukocyte count within the normal range but an elevated platelet count of 800 × 10^9^/L. These discrepancies are not captured in the current response criteria but might be of utmost importance in terms of evaluating responses in individual patients at a given time point and also when assessing the dynamics in the cell count responses over time. Such information may be even more important when considering the differences between HU and IFN in regard to inducing normal cell counts and the observed fluctuations in cell counts, which we most recently described in patients during treatment with HU.^33^

Based upon serial measurements of hematological variables (leukocyte count, platelet count, and LDH) from patients enrolled in the Danish DALIAH trial (see below), we have elaborated a novel data‐based hematological biomarker index that by a single number describes the deviation of leukocyte count, platelet count, and LDH from normal values. Using this novel biomarker index for the assessment of responses to IFN and HU, we provide evidence that IFN may be superior to HU in the treatment of patients with PV and related neoplasms. Based upon current knowledge on the mechanisms of action of HU and IFN, we discuss the rationales and perspectives for using IFN in combination with HU in the treatment of MPNs.

## 2 MATERIAL AND METHODS

The DALIAH trial is an investigator‐initiated, open‐label, randomized controlled, parallel design, clinical phase III trial (ClinicalTrials.gov Identifier: NCT01387763) conducted at eight study sites in Denmark from 2012 to 2020. In this trial, the safety and efficacy of different IFN formulations (PegIntron and Pegasys) are compared with HU. HU was administered at a starting dose of 500 to 1000 mg/day, according to the treating investigator. The dose was adjusted according to pre‐defined dose levels to achieve a hematologic response (leucocyte count <10 × 10^9^/L and platelets <400 × 10^9^/L). In order to decrease toxicity‐related treatment‐discontinuation, IFN was initially administered at low levels (Pegasys: 45 μg/week and PegIntron: 35 μg/week). The IFN dose was escalated in a response‐driven manner at pre‐specified time points to achieve a complete hematologic response at 4 and 12 months and a partial or a complete molecular response by 2009 European Leukemia Net (ELN) criteria (ET, PV, Pre‐MF) or EUMNET 2005 criteria (PMF) at 12 and 18 months.^38^, ^39^ The study was approved by the local ethical committee.

### 2.1 Calculation of biomarker index

The Hematological Biomarker Index (HBI) combines the leucocyte count, the platelet count, and LDH from the individual patient into a single number. These biomarkers have been selected since they are included in the criteria for diagnosis and treatment response (leukocyte and platelet count), and prognosis (leukocyte count). The LDH was selected as an integrated signature of myeloproliferation. The hemoglobin concentration level as well as the hematocrit level were not included in the index for several reasons. First, in patients with PV, rapid reductions in these parameters are also caused by venesection. Second, HU may induce macrocytosis unrelated to vitamin B12 or folic acid status,^17^ hence falsely elevating the hematocrit level. Third, for simplicity and usefulness of the index we aimed to minimize the number of included biomarkers.

To calculate the HBI, we determine the distance from the normal level for each of the three biomarkers (leucocyte count, platelet count, and LDH). If a measurement is above the normal level, we calculate the distance to the upper limit of the normal level and if the measurement is below the normal level, we calculate the distance to the lower limit of the normal level (see Table 1 for normal levels). If a measurement falls within the normal level, it adds zero to the HBI. To give each biomarker comparable weight, we weight the distances by the upper limit for the normal level of each biomarker. See Supplementary Materials 1, Figure 1 for an example of HBI calculation.

**TABLE 1:** Normal levels and weights are used for HBI calculations. For LDH, an average of the two age groups is taken as the inverse weight

Biomarker	Lower limit	Upper limit	Weight
Leucocytes (10^9^/L)	3.5	8.8	1/8.8
Platelets (10^9^/L)	145	390	1/390
LDH, age <70 (U/L)	105	205	2/(205 + 255) = 1/230
LDH, age ≥70 (U/L)	115	255	2/(205 + 255) = 1/230

**FIGURE 1:**
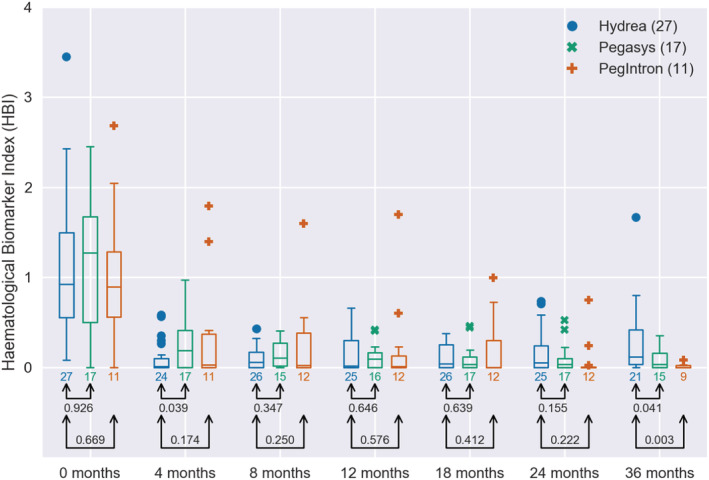
Box plots of the HBI in the DALIAH study for patients over 60 grouped by treatment. The numbers below each box indicate the number of patients. The numbers beneath the arrow pairs indicate the *p*‐value in Welch's unequal variances *t*‐test for equality of means for HU vs. Pegasys and for HU vs. PegIntron for HU vs. Pegasys and for HU vs. PegIntron.

By the relative change in *JAK2V617F* we mean (*JAK2V617F* − *JAK2V617F*_0_)/*JAK2V617F*_0_, where *JAK2V617F*_0_ is the *JAK2V617F* at baseline and *JAK2V617F* is that at the instance considered. We will use the shorthand “relative *JAK2*” to mean the relative change in *JAK2V617F*.

### 2.2 JAK2V617F analysis

Mutated *JAK2V617F* was detected by qPCR, as previously described.^40^ The sensitivity of the assay was 0.1%.

### 2.3 Statistics

**FIGURE 2:**
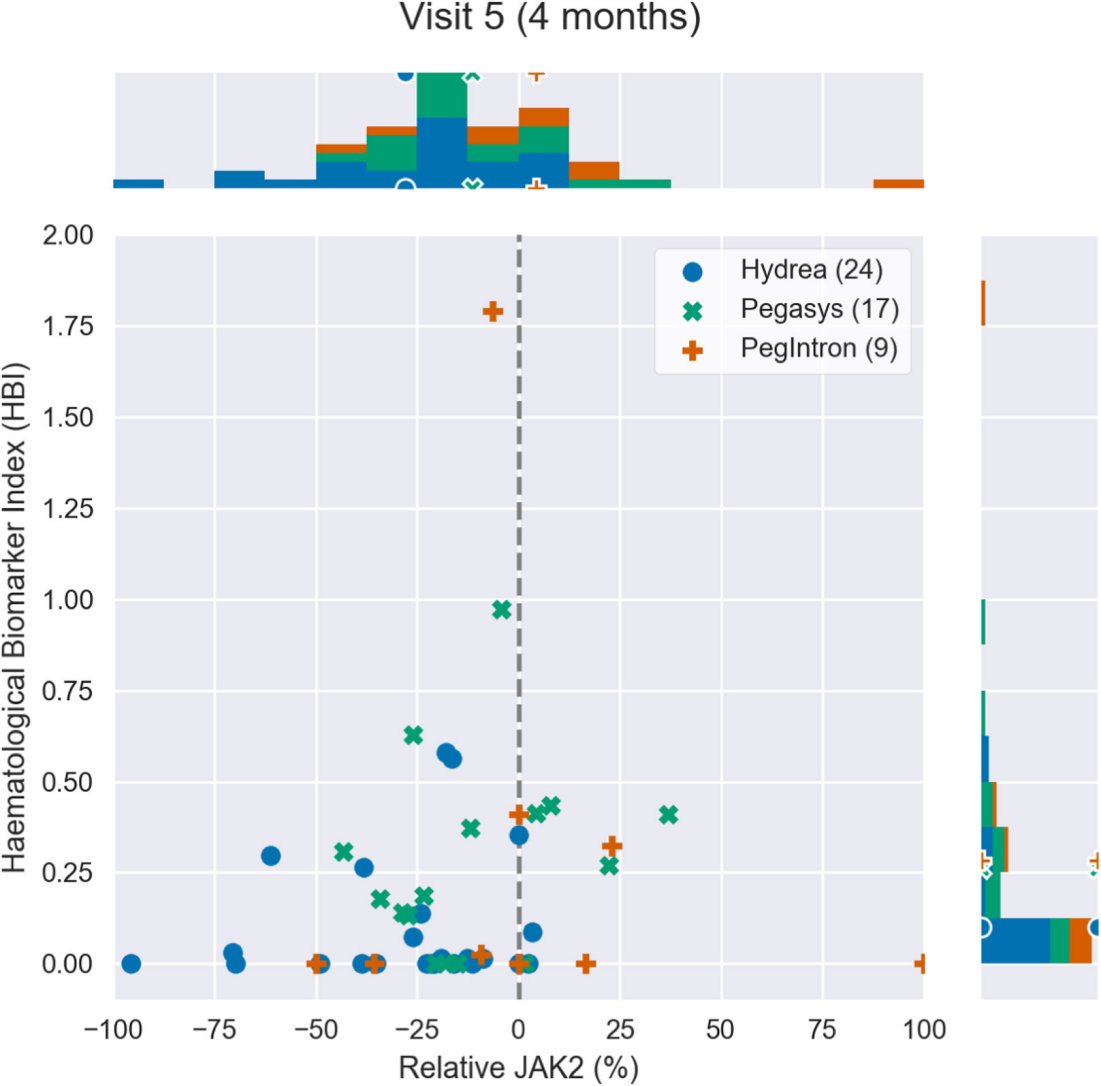
The JAK2V617F allele burden change relative to baseline versus the HBI at month 4 after treatment onset. Each point represents a patient from the DALIAH study. The legend indicates the number of patients per treatment group. Above and to the right of the main plot, the distribution of the patients is shown in a histogram. The markers (dot, star, and cross) on the histograms indicate the mean value in each treatment group. The closer a data point is to the lower left, the closer the patient is to total remission.

Computations are carried out using Python 3.5.3 and the packages Matplotlib version 3.0.3 and Numpy version 1.18.5. For statistical analysis (Figure 2), we use Python 3.5.5 and the package SciPy version 1.4.1.

**FIGURE 3:**
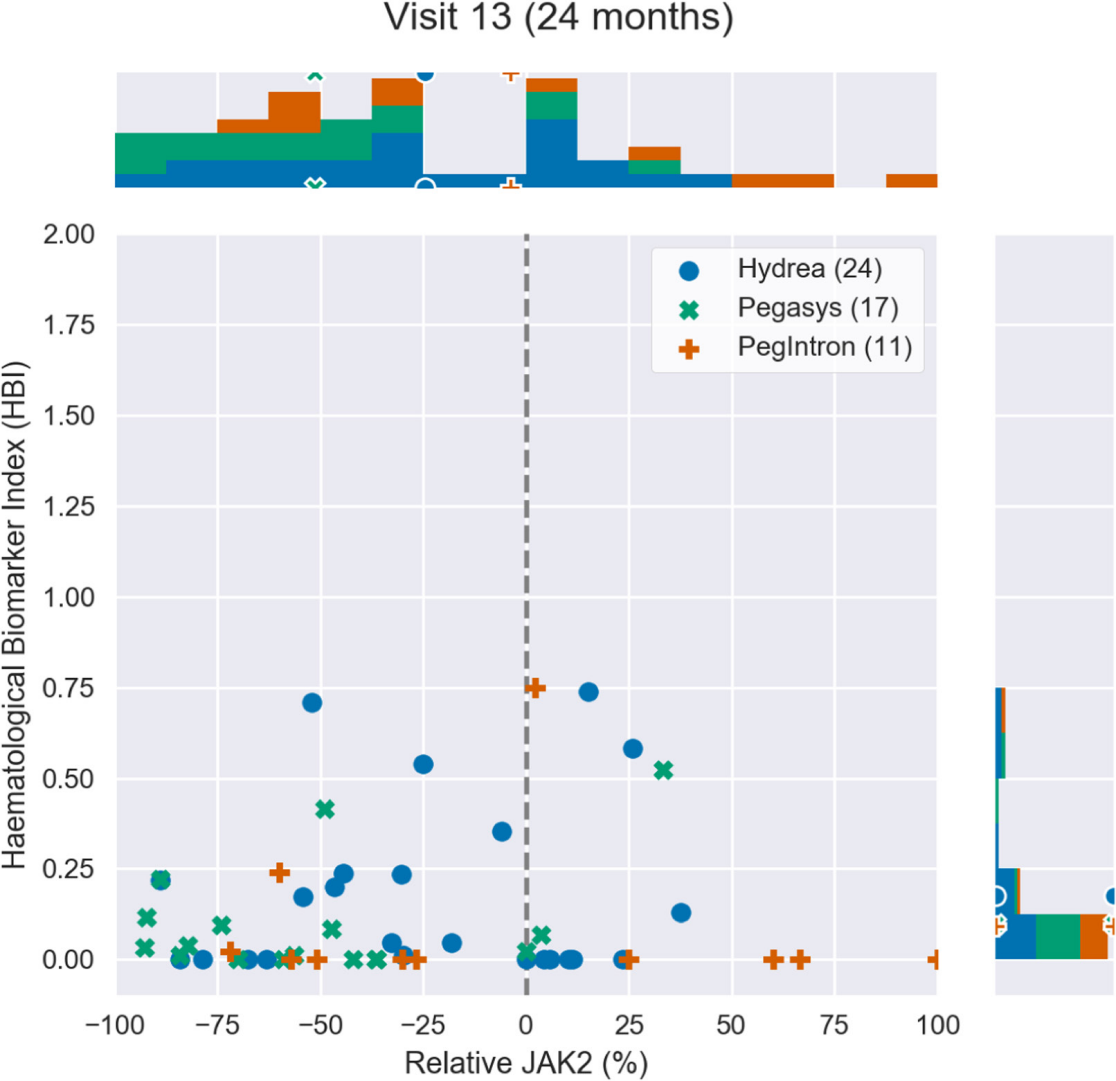
The JAK2V617F allele burden change relative to baseline versus the HBI at month 24 after treatment onset. Each point is a patient from the DALIAH study. The legend indicates the number of patients per treatment group. Above and to the right of the main plot, the distribution of the patients is shown in a histogram. The markers on the histograms indicate the mean value in each treatment group.

## 3 RESULTS

A total of 56 (ET: 12, PV: 36, Pre‐MF: 3, PMF: 3) of 206 patients, enrolled in the Danish DALIAH‐trial, were included in the study. Only *JAK2V617F* mutated patients ≥ age 60 with 5 or more serial *JAK2V617F* measurements receiving monotherapy with either HU (*n* = 27) or IFN (*n* = 29) were considered eligible. Patient characteristics by treatment group at baseline are presented in Table 2.

**TABLE 2:** Patient characteristics by treatment group

	HU, *n* = 27	Pegasys, *n* = 17	PegIntron, *n* = 13	Total, *n* = 56
MPN subtype				
ET	7 (26)	2 (12)	3 (23)	12 (21)
PV	17 (65)	12 (71)	7 (54)	36 (64)
Pre‐MF	0 (0)	2 (12)	1 (8)	3 (5)
PMF	2 (8)	1 (6)	2 (15)	5 (9)
Age (years), median (range)	69 (63–72)	66 (63–69)	65 (64–67)	67 (64–71)
Biological sex				
Female	10 (38)	8 (47)	5 (38)	23 (41)
Male	16 (62)	9 (53)	8 (62)	33 (59)
*JAK2V617F* allele burden (%)	35 (15–52)	45 (41–62)	14 (4–45)	40 (16–55)
Hemoglobin (mmol/L)	9.5 (8.6–10.2)	9.4 (8.9–11.2)	8.9 (8.1–9.5)	9.3 (8.5–10.2)
Hematocrit (vol%)	47 (43–52)	47 (45–55)	44 (41–46)	46 (43–52)
Leucocytes (× 10^9^/L)	9.9 (8.7–11.5)	47 (45–55)	44 (41–46)	9.8 (8.4–12.6)
Platelets (× 10^9^/L)	664 (552–895)	513 (361–719)	611 (380–296)	611 (431–786)
LDH (U/L)	229 (204–288)	249 (204–344)	611 (380–796)	229 (195–290)

Abbreviations: ET, essential thrombocythemia; Pegasys, pegylated interferon‐alpha2a; PegIntron, pegylated interferon‐alpha2b; PMF, primary myelofibrosis; Pre‐MF, prefibrotic myelofibrosis; PV, polycythemia vera.

Using data from the DALIAH trial, we compared the efficacy of treatment with HU and IFN (Pegasys or PegIntron) in MPN patients. In Figure 1, we plot the HBI over time for patients aged 60 and above 60 years from the DALIAH study. After 36 months, a statistically significant difference (*p* < 0.05) was recorded in the mean value of the HBI between patients treated with HU and patients treated with Pegasys or PegIntron.

**FIGURE 4:**
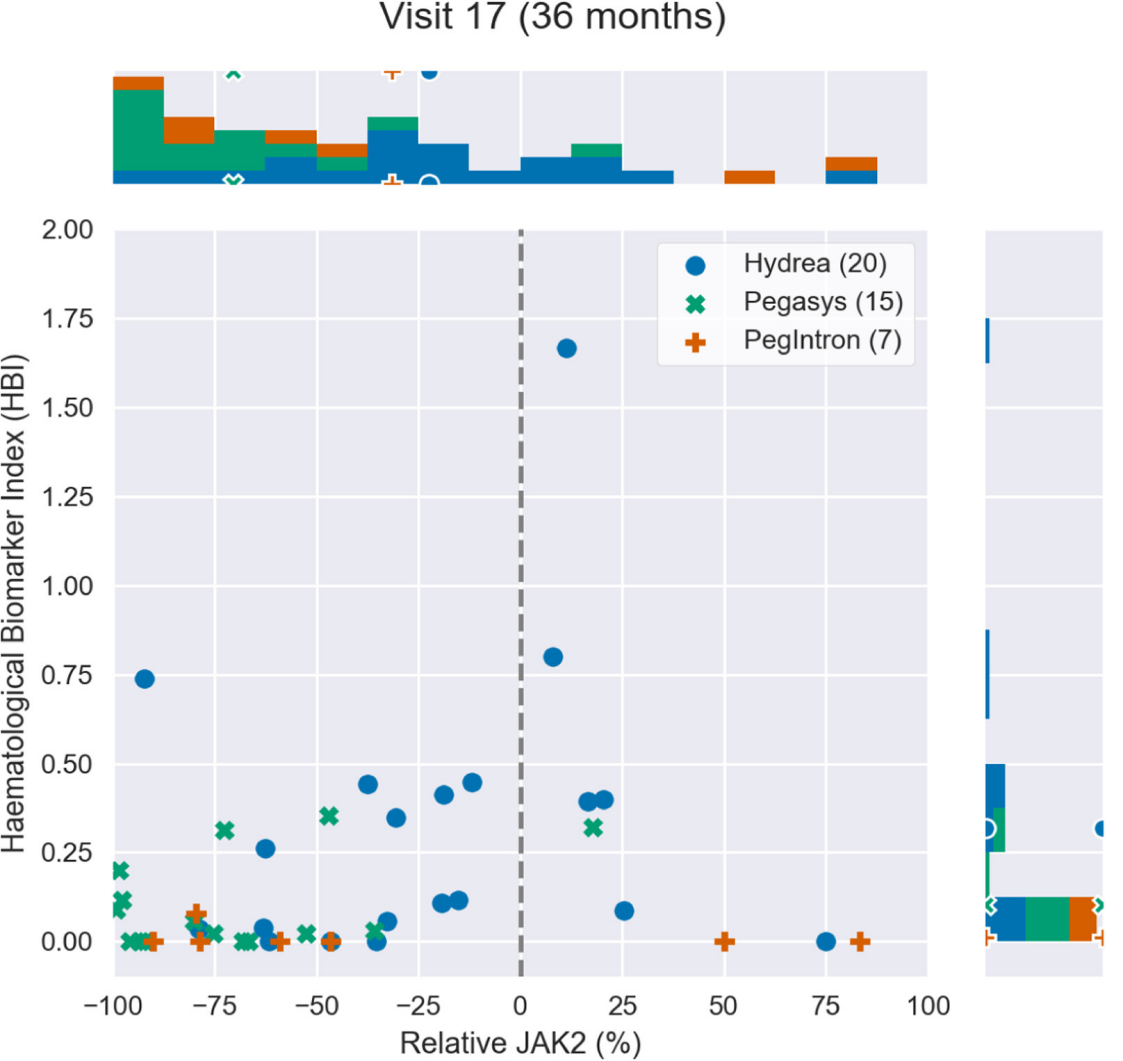
The JAK2V617F allele burden change relative to baseline versus the HBI at month 36 after treatment onset. Each point is a patient from the DALIAH study. The legend indicates the number of patients per treatment group. Above and to the right of the main plot, the distribution of the patients is shown in a histogram. The markers on the histograms indicate the mean value in each treatment group. The closer a treatment‐specific cluster of data points is to the lower left (relative to the baseline), the better effect of treatment is observed.

The molecular and hematological treatment responses are assessed by combining the HBI and the relative change in *JAK2V617F* allele burden, thus taking into account both the hematological and molecular aspects of the disease. This is shown in Figures 2, 3, 4 for 4, 24, and 36 months after treatment onset, respectively. Thus, Figures 2, 3, 4 illustrate the treatment effect over time where successful treatment corresponds to clustering in the lower left corner. We illustrate the time progression of the *JAK2V617F* allele burden and HBI as three “snapshots in time.” An illustrative animated movie of the treatment inducing partial remission over time can be found in Supplementary Materials 2, Animation 1. The three figures show that HU induces a decrease in the mean relative *JAK2V617F* allele burden early, whereas Pegasys takes effect on a longer timescale but also induces a larger change in the mean relative *JAK2V617F* allele burden. In addition, over time, patients treated with Pegasys or PegIntron achieve a lower mean HBI than patients treated with HU.

## 4 DISCUSSION

Current response criteria in MPN are based upon a combination of clinical (e.g., spleen size) and biochemical variables (blood cell counts), which are absolute values below, within, or above the normal range.^36^, ^37^ During cytoreductive treatment, some patients may at a given time point for response evaluation have obtained a normal leukocyte count but still have an elevated platelet count and vice versa. The efficacy of, for example, HU and IFN is among other things evaluated by assessing if elevated cell counts have been normalized or partially so. Thus, these efficacy evaluations do not take into account the efficacy of these agents in terms of the relative reduction in individual cell counts and their distance from the normal range, and their combined efficacy in terms of the relative reductions in individual cell counts and LDH. Such an integrated efficacy score might be a useful parameter in the evaluation of drug efficacy on cell counts and LDH.

We have herein presented a novel integrated hematological biomarker index, which over time segregated responses in individual patients and easily depicted the dynamics in their response patterns. We believe that our HBI provides a novel score that may have the potential to be used at population level to compare cohorts at baseline, compare progression of a cohort during monotype treatment, compare cohort responses to different treatments, and quantify treatment response on an individual level for longitudinal data (see supplementary for examples). Furthermore, in combination with the molecular marker, the *JAK2V617F* allele burden, the HBI provides an easily comprehensible diagram, where a patient or cohort trajectory approaching the lower left corner is the best possible outcome. Hence, this may assist in a clinical setting to help addressing whether a given treatment should be continued or altered. Furthermore, the visual nature of the diagram may be a help for a hematologist in order to communicate the patient's response status and forecast based on a personalized score on longitudinal data.

We have used HBI to compare HU and IFN from the DALIAH trial, revealing a fast but nonpermanent response of HBI to HU (initially a fast vertical downward movement in the diagram) compared to patients treated with IFN, and a much more effective *JAK2V617F* reduction for patients treated with IFN than patients treated with HU. Whereas HU has a relatively fast effect on the HBI and an indirect temporary initial effect on *JAK2V617F* followed by a relapse after approximately a year, IFN shows a slower and more steady decline with significant effects on HBI and a direct persistent effect on *JAK2V617F*. Briefly, using the novel HBI our data clearly depict that HU rapidly normalizes elevated cell counts but with fluctuating levels thereafter, while IFN works slower but with a better, more robust, and sustained result. Thus, the mathematical modeling design suggests that a combination of HU and IFN is a rational treatment option, especially within the first year after the MPN diagnosis until the efficacy of IFN has kicked in.

Only four major thromboembolic events in four patients were recorded during follow‐up; HU: 2/27 (7%) vs. IFN: 2/29 (7%). For this reason, we did not attempt to associate thrombosis with treatment group or with HBI. Also, we believe that the possible superior effect of IFN on thrombotic risk reduction compared with HU may not be evident until a longer follow‐up (> 36 months). In our study, the mean HBI was first significantly lower in the IFN groups at 36 months compared with HU (Figure 1) and the reduction of the *JAK2V617F* allele burden reduction was slower but greater with time among patients treated with Pegasys as compared with HU (Figures 2, 3, 4). In conclusion, we describe and propose a novel biomarker index for the assessment of hematological responses in patients with MPNs during treatment with HU and IFN. The novel methodology recaptures the different kinetics in response patterns between HU and IFN and the favorable long‐term efficacy of IFN. A limitation of the study includes the lack of a comparison of the individual HBI to the currently used ELN response criteria. In addition, our novel biomarker index needs to be validated in larger studies. However, the HBI seems a promising novel tool to uncover the relative responses in individual cell counts, which may yield a better signature of the “global response” in individual patients. Our data support future studies on the safety and efficacy of combination therapy with IFN and HU. These studies should include concomitant molecular, genomic, and immune cell studies to decipher in depth the impact of combination therapy with HU and IFN upon circulating CD34+ cells and progenitors.^41^, ^42^, ^43^, ^44^, ^45^, ^46^

## AUTHOR CONTRIBUTIONS

**Marc John Bordier Dam:** Formal analysis (equal); methodology (equal); software (equal); validation (equal); visualization (equal); writing – review and editing (equal). **Rasmus Kristoffer Pedersen:** Formal analysis (supporting); methodology (supporting); software (supporting); validation (equal); visualization (supporting); writing – original draft (supporting); writing – review and editing (equal). **Trine Alma Knudsen:** Data curation (lead); investigation (equal); writing – review and editing (equal). **Morten Andersen:** Conceptualization (equal); formal analysis (supporting); investigation (equal); methodology (supporting); software (supporting); supervision (equal); visualization (supporting); writing – original draft (supporting); writing – review and editing (equal). **Christina Ellervik:** Writing – review and editing (equal). **Morten Kranker Larsen:** Writing – review and editing (equal). **Lasse Kjær:** Conceptualization (supporting); investigation (supporting); writing – review and editing (equal). **Vibe Skov:** Conceptualization (supporting); investigation (supporting); writing – review and editing (equal). **Hans Carl Hasselbalch:** Conceptualization (lead); funding acquisition (equal); investigation (equal); supervision (supporting); validation (equal); writing – original draft (lead); writing – review and editing (equal). **Johnny Ottesen:** Conceptualization (lead); formal analysis (supporting); funding acquisition (equal); investigation (equal); methodology (equal); supervision (lead); visualization (supporting); writing – original draft (supporting); writing – review and editing (equal).

## Supporting information


Appendix S1

Video S1

## Data Availability

Data sharing is not applicable to this article as no new data were created or analyzed in this study.
