# Supplementary material for: A novel integrated biomarker index for the assessment of hematological responses in MPNs during treatment with hydroxyurea and interferon‐alpha2
Source: Cancer Med. 2022 Oct 17;12(4):4218–26. doi: 10.1002/cam4.5285 (PMC9972145; doi:10.1002/cam4.5285)
Supplement: Supplementary file 1 — Appendix S1 [file CAM4-12-4218-s001.pdf]

Supplementary Material, Dam et al.

The Haematological Biomarker Index (HBI) combines information about a patients thrombocyte count, leukocyte count and LDH into a single number. This is useful for comparing different treatments but also evaluating the status of a patient. To calculate the HBI we determine the distance from the normal level for each of the three biomarkers (thrombocyte count, leucocyte count and LDH), that is if a measurement lies above the normal level we calculate the distance to the upper limit of the normal level and if the measurement lies below the normal level we calculate the distance to the lower limit of the normal level (see Table 1 for normal levels). If a measurement falls within the normal level it adds zero to the HBI. To give each biomarker comparable weight we weigh the distances by the upper limit for the normal level. An example of the calculation of the HBI is shown in Figure 1. A mathematical formulation is

$$\mathrm{HBI}_i = \sum_c w_c d_c(y_{c,i}), \tag{1}$$

where $c \in \{\text{thrombocyte count}, \text{leucocyte count}, \text{LDH}\}$, $y_{c,i}$ is the i'th measurement of biomarker c, w_c is the weight given in Table 1 and $d_c(y_{c,i})$ is the difference from the normal levels given by

$$d_c(y_{c,i}) = \begin{cases} y_{c,i} - L_{c,upper}, & y_{c,i} > L_{c,upper} \\ L_{c,lower} - y_{c,i}, & y_{c,i} < L_{c,lower} \\ 0, & \text{otherwise} \end{cases}, \tag{2}$$

where $L_{c,upper}$ and $L_{c,lower}$ are the upper and lower limits for the normal levels given in Table 1.

Note that when the HBI is zero the three biomarkers are all within the normal levels and hence in treatment of patients the objective is to achieve an HBI of zero.

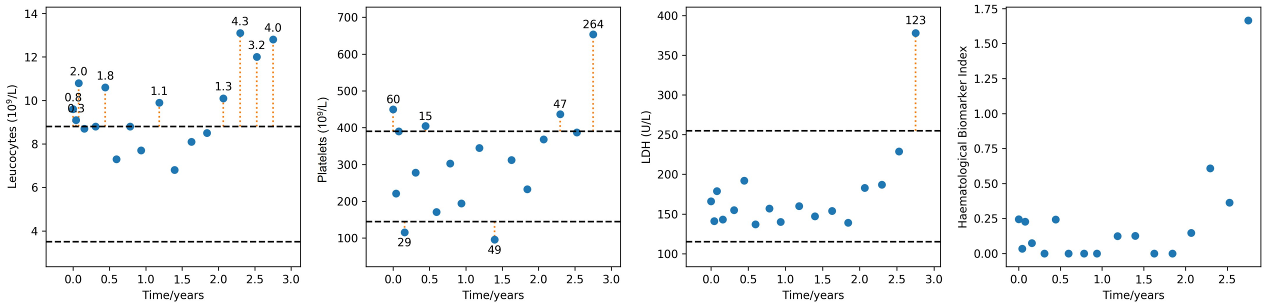

Figure 1: An example of the HBI calculation: The numbers indicate how far from the normal level each measurement is. The measurements which fall inside the normal levels do not contribute to the HBI. Taking the final measurement as an example the thrombocyte contribution is $264/390 \approx 0.68$, the leucocyte contribution is $4.0/8.8 \approx 0.45$ and the LDH contribution is $123/230 \approx 0.53$ and thus the HBI in this case is approximately $0.68 + 0.45 + 0.53 = 1.66$.

Table 1: Normal levels and weights used for HBI calculations

Biomarker (c)	Lower limit ($L_{c,lower}$)	Upper limit ($L_{c,upper}$)	Weight (w_c)
Leucocytes (10^9/L)	3.5	8.8	1/8.8
Platelets (10^9/L)	145	390	1/390
LDH, age < 70 (U/L)	105	205	$2/(205+255) = 1/230$
LDH, age ≥ 70 (U/L)	115	255	$2/(205+255) = 1/230$
